# Close2U: An App for Monitoring Cancer Patients with Enriched Information from Interaction Patterns

**DOI:** 10.1155/2020/3057032

**Published:** 2020-07-15

**Authors:** Javier Navarro-Alamán, Raquel Lacuesta, Iván García-Magariño, Jesús Gallardo, Elena Ibarz, Jaime Lloret

**Affiliations:** ^1^Department of Computer Science and Engineering of Systems, University of Zaragoza, Zaragoza, Spain; ^2^Instituto de Investigación Sanitaria Aragón, University of Zaragoza, Zaragoza, Spain; ^3^Department of Software Engineering and Artificial Intelligence, Complutense University of Madrid, Madrid, Spain; ^4^Instituto de Tecnología del Conocimiento, UCM, Madrid, Spain; ^5^Departamento de Ingeniería Mecánica, Universidad de Zaragoza, Zaragoza, Spain; ^6^Integrated Management Coastal Research Institute, Universitat Politecnica de Valencia, Valencia, Spain; ^7^School of Computing and Digital Technologies, Staffordshire University, Stoke, UK

## Abstract

The management of cancer patients' symptoms in doctor consultations is a cornerstone in clinical care, this process being fundamental for the follow-up of the evolution of these. This article presents an application that allows collecting periodically and systematically the data of cancer patients and their visualization by the medical team. In this article, we made the analysis, design, implementation, and final evaluation by analyzing the correlation of this data collection with interaction patterns to determine how the user information can be enriched with information from the interaction patterns. We have followed an agile methodology based on the iterative and incremental development of successive prototypes with increased fidelity, where the requirements and solutions have evolved over time according to the need and assessments made. The comprehensive analysis of the patient's condition allowed us to perform a first analysis of the correlation of the states of patients concerning mood, sleeping quality, and pain with the interaction patterns. A future goal of this project is to optimize the process of data collection and the analysis of information. Another future goal is to reduce the time dedicated to reporting the evolution of symptoms in face-to-face consultations and to help professionals in analyzing the patient's evolution even in the period that has not been attended in person.

## 1. Introduction

The management of the symptomatology of patients consumes more than half the time spent by health professionals in monitoring the patient's evolution. It is considered a cornerstone of clinical care, particularly for patients with chronic diseases. However, symptoms and physical disabilities are not detected by health professionals until the patient has an appointment. As a result, the opportunity to intervene and alleviate suffering is lost. In addition, the incomplete information in the Electronic Health Records (EHRs) limits the ability of medical professionals to understand the results of patients. There are several mHealth applications that can remotely monitor patients to overcome this barrier in many medical fields, such as the app for monitoring patients with risk of suffering cerebral strokes by means of cloud services [1]. There are systems based on wearables that also monitor patients, like the wireless body area network system for controlling obesity [2]. There are proposals for monitoring patients with 5G networks, such as the architecture and protocol for smart eHealth monitoring [3].

The number of people who suffer and overcome cancer is continuously increasing, so there is a great concern for their quality of life and quality of care. Most of these people adapt well psychologically and physically after completing their initial treatment. Thus, it is essential to identify the needs in progress to provide support as soon as possible. The need of monitoring oncological patients is considered a fundamental aspect in their follow-up [4], the main objective being the maximization of the benefits of treatments adjusted to the effects and reactions. The impact of knowing the disease greatly influences the roles played by patients at home, at work, and in their community [5].

The articles [6, 7] agree that there are usability problems in health applications. Therefore, the participation of patients in the design is very important, so that it can have an impact on the state of patients through the use of the app. According to [6], patients are interested in using these new technologies, but current tools are not suitable for them. For this reason, we aim at analyzing the data and give patients control over the collection of their data and see how their status affects the use of the application.

In this work, the end users are cancer patients, being the evaluation of all the possible influential factors on their evolution (psychic, physical, treatments, and pharmacology) fundamental. For a better design and development of the application, we follow a patient-centered design, with patients and medical professionals in successive interviews with them.

The objective of the present study is to perform an integral follow-up of cancer patients through the use of a mobile application that allows registering the levels of physical and psychological follow-up, as well as additional influential parameters in its evolution: treatments, medication, activities carried out, and quality of sleep among others. This monitoring allowed professionals to evaluate the effectiveness of the treatments, as well as to guide or personalize their selection, identifying additionally the population at risk and thus improving the quality of life of patients.

Additionally, parameters are recorded to take interaction patterns into account, such as the choice changes made by the patient when replying to each question and the whole reply time. Through the study of the collected data, we made an analysis of the relationship between user variables (concerning mood, sleeping quality, and pain) and the interaction patterns.

The rest of the paper is structured as follows. The related work is studied in Section 2.  Section 3 analyzes the materials and methods that we use in this research. The results of the research are presented in Section 4.  Section 5 discusses the results and the limitations. Finally, the conclusion and future work are mentioned in Section 6.

## 2. Related Work

The Human-Computer Interaction (HCI) applied to the field of health is increasingly essential in the development of interactive systems for medical professionals and patients [8]. The work of medical professionals (doctors, psychologists, etc.) can be simplified by the continuous use of technology to obtain information in real time from patients. Patients can be supported by technology during their treatment or recovery process, both in the diagnosis and treatment phases, and in the case of having successfully overcome the disease. Providing patients with these applications can be beneficial for them as they are more pleasant, or at least not as monotonous, in their treatment of the disease. In addition, one can motivate them to complete their daily tasks and their treatment such as daily activities and medication. One of the main objectives is to improve the monitoring of patients with the aim of also improving the quality of life of people. For this purpose, the HCI discipline contributes to the design of usable interfaces that improve the record of their evolution.

The analysis of emotions has begun to acquire scientific interest according to the possible applications, increasing the number of studies related to the emotional component and its form of evaluation in different areas [9].

The role of emotional communication in the context of HCI is very relevant and challenging when considering the different areas of technological application [10], like in the example that focuses on remote medical care and assistive technologies. In this area, there is an increasing interest in this type of research, which includes a synthesis of the effects and emotion. Emotions affect human behavior and the system, so when a user is using an application, their emotional state can affect the usability of the system [11].

The term mHealth is defined as the union of mobile computing, medical sensors, and communication technologies for health care [12]. MHealth is an upgrowing field about the application of mobile technologies in health, which in recent years has emerged as an important segment of telemedicine and its main objective is to improve health services, integrating the benefits of mobility and ubiquity, typical of mobile systems, to the care treatments of traditional health, pretending to bring health care to people and not people to the health system. MHealth applications are creating mechanisms for the exchange of information related to health care, even in remote and low-income areas, due to the large area of coverage and social influence of mobile telephone networks, becoming a factor strategic to save lives [13]. In this context of mHealth, we will focus on the follow-up of cancer patients.

In addition to the intervention of the doctors in the consultations with their patients, another relevant aspect is the optimization of their next consultations before doing them. In this way, they can improve the way in which the medical professionals dictate a new treatment. There is an application of medical treatment to optimize the consultations of medical patients according to the known preferences and other selection criteria [14]. That work refers to optimizing the appointments of the patients, the insurance payment options, the treatment in the medical facilities, and other aspects of the interactions between patients. The application gathers data from the patient's history and calculates a placement score in a medical center based on a plurality of parameters of the user's medical history associated with the user's medical history and schedules the medical care consultation at a medical center that provides the highest placement score in a medical center. Many more applications are being developed and can be downloaded through the Google Play Store for Android phone users. Some of the mHealth systems are for some hereditary diseases and disorders [15], cancer-based apps [16–19], or studies on the impact of stress or emotions on the interaction with the application [11, 20].

Android Mobile Informatics Application for Some Hereditary Diseases and Disorders (AMAHD) is a complementary framework for medical practitioners and patients. The mobile application will help to sensitize and complement the efforts of biomedical, medical, and bioinformatics researchers working in the areas of inheritance research and genetics. AMAHD has proven to be a valuable resource for the research company in the battle against hereditary diseases and disorders [15].

There are some cancer-based applications. For instance, a smartphone-based pain management app for adolescents with cancer provides patients pain management support based on their individual pain [16]. Another article evaluates the usability of their app and shows the results of the iterative development of their app. Its authors inform other developers and researchers in development, integration, and evaluation of mobile health apps and services that support cancer patients in managing their health-related issues [17]. The goal of another app is to stabilize a daily functional activity in breast cancer patients. App-using participants could more frequently report adverse events, and those under supervision made fewer and more precise entries than unsupervised participants [18]. The last reviewed app is a smartphone app framework for segmented cancer care coordination, which provides both medical risk assessment and health care monitoring functions [17]. However, none of these apps considered information from interaction patterns to enrich the extracted information, as the current work does.

Besides implemented applications, some papers have studied the impact of emotions over the medical applications and how emotions can be related to the usability of the system that they are interacting with [11, 20]. Defining the stress level of the user can train the system in such a way that it could not only detect the user's stress level but can also modify itself accordingly, thereby increasing the usability of the system and the user satisfaction [11]. In [20], it is shown that experiencing negative emotions during the use of the system can negatively influence important user behaviors, including the client's decisions regarding the application.

In conclusion, monitoring and reminder applications have been helpful for users in assisting them in monitoring and keep track of the patient's health care records as well as their medication intake, and there are some studies that show how the emotions can affect the usability of mobile applications. This motivates the development of Close2U application with the intention to help the medical practitioners with the analysis that the app can bring them and help them in improving the treatment for the patient. In addition, a novelty of Close2U app is that it shows that the analysis of interaction patterns can enrich self-reported user information. This work analyzes the correlations among cancer-patient variables and interaction-pattern variables.

## 3. Materials and Methods

### 3.1. Materials: Close2U App

The main material for this research is the mobile application Close2U. The application contemplates the integral monitoring of patients. Patients were able to see and manage both their medication and their appointments. For the part of the appointments that have both with the psychologist and with the doctor, a section was designed in which they can visualize the appointments in a calendar or in a detailed list, and they can be managed by them. The part of the patient's medication has also been designed, in this way they can keep track of his prescriptions, allowing them to visualize and manage them themselves; the medication can be viewed weekly or through a list with the details.

The methodology used for the application Close2U was an agile methodology based on the iterative and incremental development of successive prototypes with increased functionality. The application has evolved in its phases of analysis, design, implementation, and evaluation. The successive refinements have allowed the evolution of the prototypes and the increase of their functionality and quality. The agile methodology may be the most appropriate for projects that suffer a high number of changes and need more control and communication with the client in real time and allows both adapting to problems that may arise and making the necessary changes at the beginning of each phase, without having to wait to finish all the actions.

Nowadays, it is crucial to incorporate the user into the design and implementation cycle of a mobile application. In addition, in the medical-scientific research community, there is a growing thought that states that the control and prevention of cancer must incorporate a communication with the patient [10]. In this way, the benefit of current medical discoveries in diagnosis and treatment is maximized, particularly in the emerging era of personalized medicine. Although patient-medical communication has focused on results such as patient satisfaction, understanding, and assessment, we must strengthen the understanding of how these can impact on their attitude and the results of the disease [10]. Throughout all phases of development, we have worked in cooperation with the psychologists of the AECC (Spanish Association against Cancer) in Teruel, who were present throughout the cycle of design, implementation, and evaluation of the product.

To design and develop the application, a conceptual framework was first made; then the necessary algorithms for data capture were designed and the software tools necessary to design it were selected. The developed application allows one to treat and use all the information collected about physical condition, activities, treatments, and so on. Each element of the follow-up was fundamental to know the overall situation of the patient, maintaining their privacy. Information was collected and, in order of priority, actions to be carried out were defined.

#### 3.1.1. User Interface of Close2U App

This section presents the user interface showing the most relevant screens of the final implementation of Close2U app. The changes that occurred in the successive evaluations will be explained, since changes were made throughout the implementation, with the help of the evaluation we made together with the psychologists and the cancer patients.

**Figure 1:**
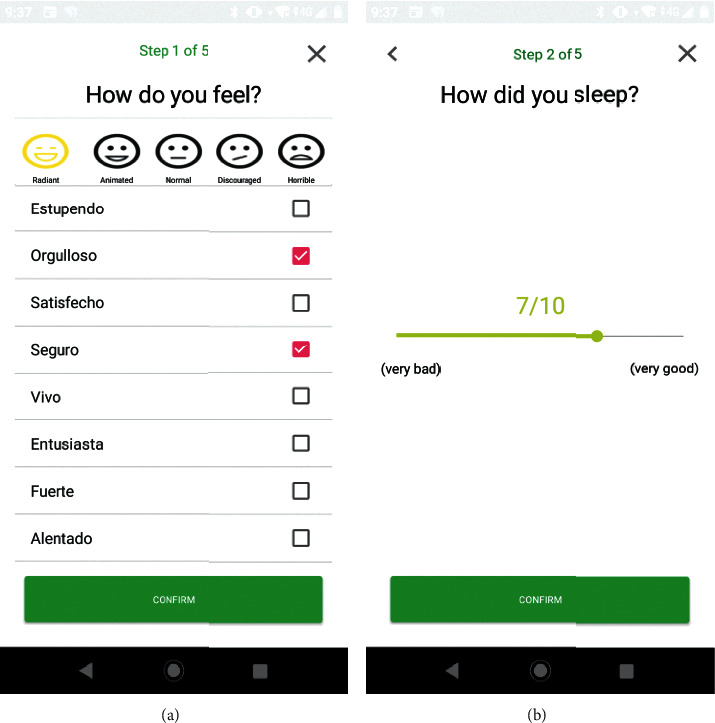
Screens of the user interface 1: (a) mood selection and (b) sleep quality selection.

Figure 1(a) shows the screen in which the patient is asked about their mood. It is a question that had many changes since at the beginning, the emotions were not taken into account, only the mood, which limited the patient to specifying his mood. Once the emotions were introduced, it was possible to clarify in more detail how they feel, opening the way to a new “mood register” called “Undecided”; this state of mind depends on the emotions that the patient selects. When the patient selects various moods, the medical professional can see the situation of patient's indecision.

**Figure 2:**
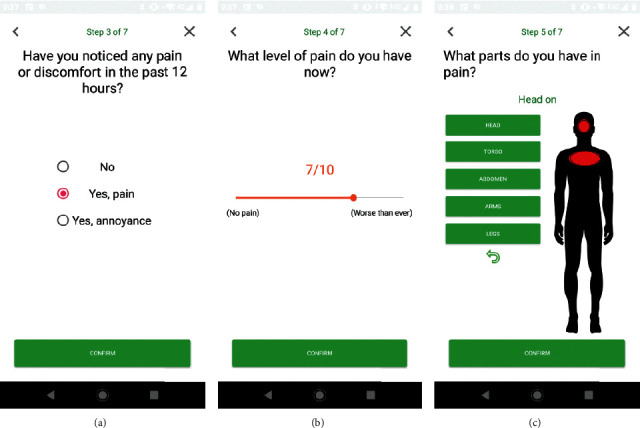
Screens of the user interface 2: (a) pain/discomfort selection, (b) pain level selection, and (c) body parts with pain selection.

In the screen of Figure 1(b), one can see the question about sleep quality. This screen was modified several times according to the requirements and evaluation meetings established by the psychologists of the AECC Teruel; it was decided to show the patient in a numerical form from 0 to 10, since it is more comfortable and clearer in terms of usability. This same change was also made with the level of pain, which is presented later in Figure 2(b).

Figure 2(a) shows the options to assess whether you feel pain or discomfort, or do not feel both. Initially only the pain was registered, but later considering the comments from patients and psychologists, it was later decided to add a differentiation between pain and discomfort.

Another valued aspect was the parts where the patient felt pain or discomfort, and a screen was made with which the patient could visualize a body in which a pain could be indicated by buttons to make it more visual. This is intended to be more intuitive for the patient as seen in Figure 2(c), since at first there was no image but simply buttons.

#### 3.1.2. Internal Functioning of Close2U App

The purpose of the design phase was to ensure that the developed application meets the requirements of the end user before the prototype is translated into the production application. We used the Unified Modeling Language (UML) for defining the design diagrams.

**Figure 3:**
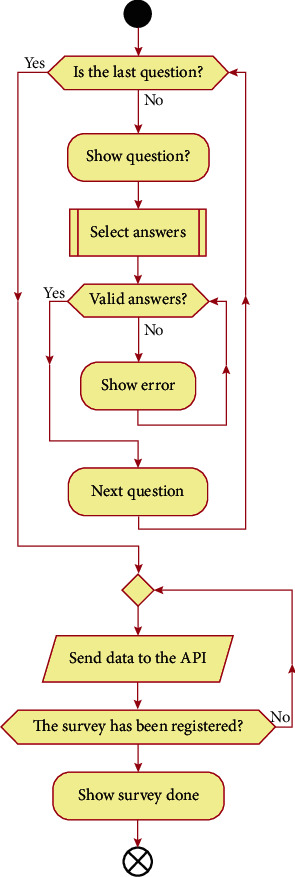
Activity diagrams of Close2U app.

To carry out the plan of user activities, the psychologists of the AECC Teruel were taken into consideration. In this way, the interactions that the user must perform with the application were identified.  Figure 3 shows how patient interacts with the application when carrying out the surveys. In each question, the app checks that the answer is valid and continues to the next question until the end of the questionnaire. The replies are sent through the API, and it will be verified that the survey has been registered. In the user interface, the app confirms the patient that the survey has been successfully registered.

For the selection of the questions to be shown in the survey, a previous analysis was carried out and they were refined through successive evaluations. Next, the interface associated with each of them was designed, as described in Section 3.1.1. For this stage, the flow of activities and their order were designed, so that in this way the patient will “connect” better with the survey and neither feels bad when doing it nor gets bored along with it.

**Figure 4:**
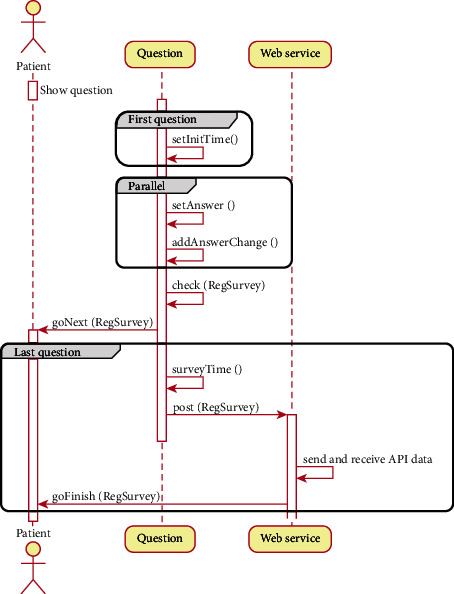
Sequence diagram of Close2U app.

In the next stage of the design, once the activities were defined, a sequence diagram was defined, and Figure 4 presents this diagram, in which we show how the surveys work internally in the application. For all the questions shown on the screen, in parallel when the patient answers each question, the change is added to that answer. Then, when the patient confirms their answer, it goes to the next question. For the first and last questions, there are special sequences. In the first question, the start time of the survey is recorded and in the last question the total time is calculated, the response is sent to the server, and finally, the survey ends.

At the sequence diagram, the objects are shown as lifelines along the survey and with their interactions over time represented as arrows from the origin lifeline to the destination lifeline. Sequence diagrams are appropriate for showing which objects communicate with each other and the messages that trigger those communications.

For the implementation, the IDE (Integrated Development Environment) Android Studio was used, together with its emulator to perform the patient part. The implementation and validation were carried out over twelve months.

The validation of the application has been made from the requirements analysis phases until the final implementation with the medical professionals of the AECC Teruel. The first tests with patients allowed us to correct the existing conceptual and design errors.

Once the first functional prototype of the application was ready, tests were carried out with the users. Based on them, we got back to the design phase and improved the design. Through the evaluation carried out, the interfaces were modified to improve aspects of the usability and functional application, for example, so that this would be intuitive for the patient. In addition, being able to assess the mood of the users during the use of the live system had a practical meaning for the design and improvement of our application.

### 3.2. Methods

#### 3.2.1. Participants

There were 23 users that voluntarily participated in this study, without any economical compensation. They were 50.21 years old in average with a standard deviation (SD) of 10.09. They were 6 males and 17 females. The sample included 7 cancer patients and 16 healthy people.

#### 3.2.2. Measures

We evaluated sleep quality with the self-reported question “How did you sleep?” replied in a 0–10 range (zero meaning the worst sleep quality and ten meaning the best). We determined the existence of pain with the question “Have you noticed any pain or discomfort in the last 12 hours?” with an answer from “yes,” “no,” and “discomfort” options. If the user replied affirmatively, then the app asked, “What level of pain/discomfort do you have now?” which was answered in a 1–10 range (one, minimum pain level and ten, maximum). The app recorded zero level if the user had previously answered to feel neither pain nor discomfort. We evaluated the mood by asking the user “How are you feeling?” with a multiple response among 97 moods classified in five mood categories (1: horrible, 2: discouraged, 3: normal, 4: animated, and 5: radiant), categorized by the medical professionals with who we worked in every step of the design and development of the app. These moods were the following ones:

- Category 1: overwhelmed, agitated, ashamed, depressed, angry, exhausted, hurt, scared, alone, apprehensive, defeated, desperate, despondent, exhausted, helpless, angry, frustrated, impatient, pessimistic, self-critical, irritated, defensive, despised, and resentful
- Category 2: guilty, stressed, angry, bad, grumpy, dizzy, nervous, sad, discouraged, apathetic, worried, anxious, agitated, hurt, disappointed, distressed, nostalgic, offended, hungry, lacking confidence, impotent, repentant, insecure, and rejected
- Category 3: bored, confused, asleep, busy, pensive, tired, neutral, unequal, confused, hesitant, lazy, disconnected, reserved, indifferent, apprehensive, conflicted, disconnected, reserved, indifferent, scattered, restless, sensitive, and vulnerable
- Category 4: well, happy, awake, relieved, cared, empathetic, not critical, confident, calm, and sincere
- Category 5: super, proud, satisfied, confident, alive, enthusiastic, strong, encouraged, excited, grateful, hopeful, and open-minded

We labeled the responses with more than one mood as undecided. We assessed the indecision for each question with the number of times that the user changes their reply before submitting it. We applied this measure to (a) sleep quality, (b) whether the user felt pain, (c) the level of this pain, and (d) the mood.

We used System Usability Scale (SUS) [21] to measure the usability of the app.

#### 3.2.3. Protocol

Firstly, we followed a user-centered design. Three psychologists familiar with cancer tested the Close2U app during the whole development process and provided feedback. The developer improved the app following their recommendations until they were satisfied, to achieve a high level of usability. In this way, the functionalities of the app were enriched according to the common specific needs of cancer patients and their doctors.

The app was uploaded to Google Play, which is the main store of apps for Android. We encouraged cancer patients to use the app in a noncontrolled environment, advising them to use the app regularly.

We measured the collected information about users (related to mood, sleep, and pain) and the information from interaction patterns (number of changes when selecting replies to specific questions and the global survey time). We analyzed the correlations among all these variables.

We measured the usability of the app at the end of the study with SUS scale.

## 4. Results

Table 1 shows (a) the average of the changes made by the patient each time he had to choose the emotions that mark his mood and (b) the time the survey took.

**Figure 5:**
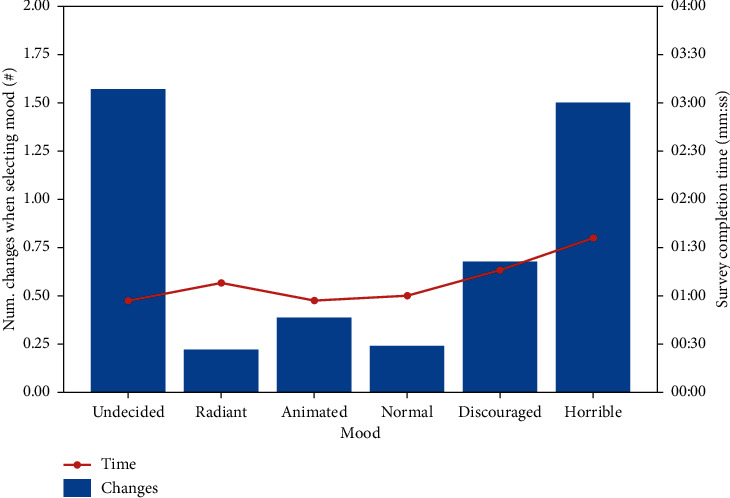
Relation of mood changes and time of realization.

In Figure 5, it can be observed that the patient modifies his response in a greater number of occasions when his mood is negative. When the users are undecided, they usually take more changes before selecting their final state. Also, in this case, they select more than one state of mind. The average of the number of changes in this case is 1.57. Regarding the total time of the survey, this was generally longer when the patient felt worse.

In a first approximation, it can be estimated that the indecision corresponds to positive moods, since the average completion time of the undecided survey was similar to the times of positive states.

Table 2 shows the average of the changes made by the patient each time he had to choose the level of pain and the time in which he performs the survey.

**Figure 6:**
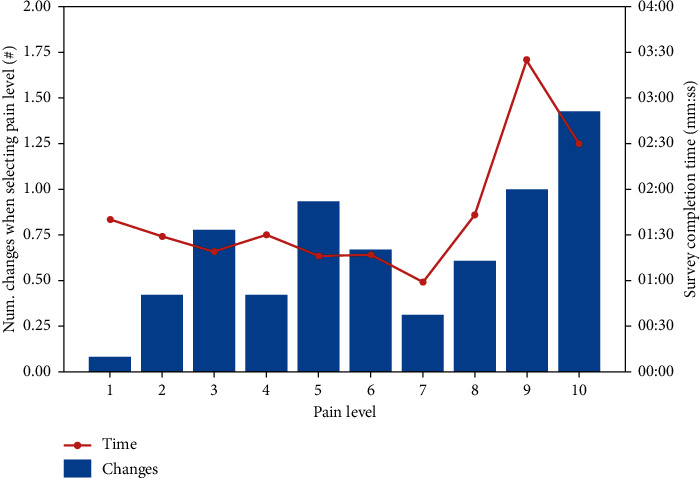
Relation of pain level changes and time of realization.

What we can observe in the graph of Figure 6 is an increase in the number of changes that the patient made when they had more pain, as in the time of conducting the survey that is higher when the patient chose a higher level of pain. This relation was also observed in other experiments, as one can see in the next presented graph.

Table 3 shows an average of the changes made by the patient when he had to decide if he had pain or not, and in the case of having pain if it is discomfort or pain and the time in which he performed the survey.

**Figure 7:**
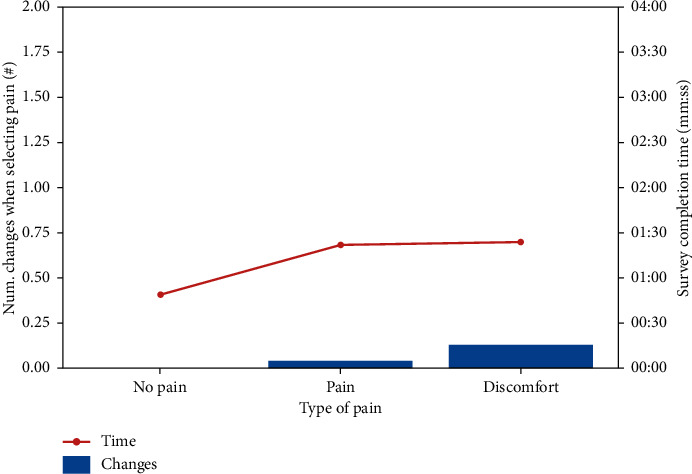
Relation of pain changes and time of realization.

In the graph of Figure 7, it can be seen to what extent it affected that the patient did not have pain, and we found that it took less than half the time to complete the survey. When comparing pain and discomfort, there were not such differences in the time to carry out the survey.

**Figure 8:**
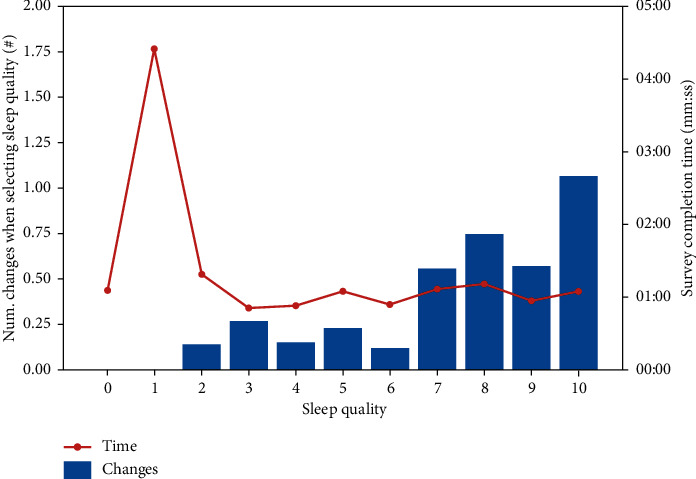
Relation of sleep quality changes and time of realization.

Table 4 shows an average of the changes that the patient makes when deciding the level of sleep and the time in which the survey is performed, for each sleeping-quality level.  Figure 8 shows this information graphically. One can observe that with the exception of a peak in level 1, the time of conducting the survey barely changed. By contrast, the number of changes was higher when the patient had slept better.

What we can observe in the graph of Figure 8 is that, with the exception of the peak that exists in level 1 of sleep, the time of accomplishment of the survey hardly changed. On the other hand, the number of changes increased as the patient had slept better.

In order to determine if there were statistically significant correlations, we conducted the Pearson correlation test between user variables (i.e., mood, sleeping quality, the existence of pain, and pain level) and the number of changes when selecting these variables.  Table 5 shows the results of this test. We considered 497 cases (considering a case each time a user performed the complete survey in the app) for this analysis. We considered 2-tailed significances as we did not know whether the correlations would be direct or inverse beforehand.

Regarding the user variables and interaction patterns concerning the numbers of changes, the test found significant correlations in the pairs (a) mood and number of changes when selecting mood, (b) sleep and number of changes when selecting sleep, (c) pain and number of changes when selecting pain, (d) pain and number of changes when selecting pain level, (e) pain level and number of changes when selecting mood, (f) pain level and number of changes when selecting sleep, (g) pain level and number of changes when selecting pain, and (h) pain level and number of changes when selecting pain level. Concerning the user variables among each other, the test found significant correlations in all the possible pairs among the user variables (1) mood, (2) sleep, (3) pain, and (4) pain level. All the significant correlations between mood-related variables and other variables had a negative correlation coefficient. However, all the significant correlations among non-mood-related variables had a positive correlation coefficient.

In order to determine if the correlations between survey time and user variables were significant, we conducted Pearson's correlation test, and Table 6 shows the results. This test found significant correlations between (a) survey time and (b) the pain-related variables including pain and pain level.

**Figure 9:**
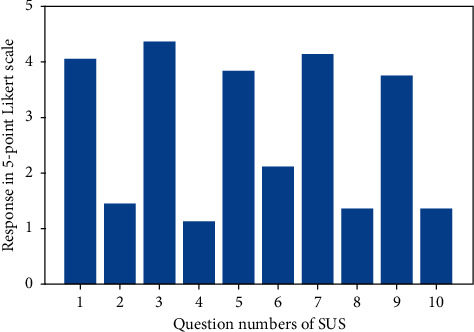
Results of the SUS scale.

Regarding usability, the average result of SUS test was 69.2 in the standard range of 0–100, and the standard deviation (SD) was 20.0.  Figure 9 shows the results of the individual items from the SUS scale.

## 5. Discussion

We detected a correlation between mood and the number of changes when selecting the answers. This reveals that the number of changes when selecting mood is relevant as it is correlated with useful information. This implies that cancer apps can collect enriched information from users by counting the number of changes when selecting a response in questions. Therefore, interaction patterns provided relevant information concerning moods.

The results revealed that users were doubtful (i.e., they changed the replies more times) when selecting (a) negative moods, (b) high-quality levels of sleep, and (c) high levels of pain. Notice that we distinguished between low and high values for each user variable, and this was concluded from the sign of correlation coefficients. For example, in the case of moods, this may reveal that users perceived negative emotions as a combination of some basic emotions, in accordance to the theory of Ekman [22] about basic emotions, rather than for positive emotions.

Another relevant finding was that pain level was significantly correlated with all the other studied variables including the user variables and the numbers of changes when selecting any user variable. Hence, pain level was the variable with most relevant information due to its correlations with all the other variables.

In the significant correlations, the sign of correlation coefficients showed that mood-related variables were inversely correlated with sleep-related variables and pain-related variables. The inverse correlation between mood and sleep variables makes sense because the pain negatively influenced on the mood of users, as the pain probably did not let them sleep well.

The correlation between the survey time and the pain (including the existence and its level) revealed that the interaction patterns also provided useful information about pain. The survey of the app dedicated a large part to the pain, since the user was asked about the specific locations of the pain in a silhouette-based interface. Users spent more time depending on when they had a higher level of pain. Thus, future applications for cancer patients with silhouette-based interfaces could indirectly help in estimating the pain level.

One of the limitations of this research is the small sample size of participants. This sample size only allowed us to detect correlations with large effect sizes. Thus, we may have not detected correlations with small or medium effect sizes.

## 6. Conclusion and Future Work

A mobile application has been developed that performs a complete follow-up of the patient. This article has focused on the self-reported moods, sleeping quality, and pain when the patient is using our application, to provide a tool for psychologists to (a) improve their treatment, (b) improve appointments with them, (c) carry out a daily monitoring of patients, and (c) be able to communicate with them outside face-to-face consultations. This increased the patients' sense of being taken care of daily, since they did not feel alone.

This article shows (1) how a mobile health application can assist patients in being more active in managing their care, (2) how the emotions affect when completing the survey, and (3) taking more time or making more changes usually depend on their mood, pain, or sleep quality at that time. Our research revealed that interaction-patterns variables provided useful information from some user variables, proved with the statistically significant correlations detected in this study.

This work motivates future research concerning the extraction of implicit information from interaction patterns in cancer monitoring apps. The correlation between user variables and interaction patterns triggers a promising research line about getting patients' information from their patterns when interacting with apps, being able to enrich the collected information with this non-self-reported information. This research could also lead to take implicit-extracted mood to adapt the interface to the needs of patients.

According to medical professionals, the app helped them to collect relevant data about the disease more easily than if they had had to collect these manually. In the future, we plan to analyze how treatment improves and how waiting times are reduced before face-to-face consultations.

In the future, it is intended to perform the noninvasive recording and measurement of the evolution of patients, by means of devices or sensors, to improve both their treatment and their quality of life. For this purpose, we will search available devices or sensors that will support the capture of evaluable parameters that affect patients, at the same time that our application will collect data. Initially, smart sensors and wristbands will be used to record the heart rate and the Electrocardiograms (ECG), blood pressure, and Heart Rate Variability (HRV). We will also explore the registry of other parameters such as oxygen level in the blood, skin and body temperature, and breathing patterns among others.

## Figures and Tables

**Table 1:** Relation of mood changes and time of realization.

Average	Undecided	Radiant	Animated	Normal	Discouraged	Horrible
Num. changes when selecting mood (#)	1.57	0.22	0.39	0.24	0.67	1.50
Survey completion time (mm:ss)	00:57	01:08	00:57	01:00	01:16	01:36

**Table 2:** Relation of pain level changes and time of realization.

Average	1	2	3	4	5	6	7	8	9	10
Num. changes when selecting pain level (#)	0.08	0.42	0.78	0.42	0.93	0.67	0.31	0.61	1.00	1.43
Survey completion time (mm:ss)	01:40	01:29	01:19	01:30	01:16	01:17	00:59	01:43	03:35	02:30

**Table 3:** Relation of pain changes and time of realization.

Average	No pain	Pain	Discomfort
Num. changes when selecting pain (#)	0.00	0.04	0.13
Survey completion time (mm:ss)	00:49	01:22	01:24

**Table 4:** Relation of sleep quality changes and time of realization.

Sleeping quality	0	1	2	3	4	5	6	7	8	9	10
Num. changes when selecting sleep quality (#)	0.00	0.00	0.14	0.27	0.15	0.23	0.12	0.56	0.75	0.58	1.07
Survey completion time (mm:ss)	01:06	04:25	01:19	00:51	00:53	01:05	00:54	01:07	01:11	00:57	01:05

**Table 5:** Pearson's correlation test between user variables and number of changes when selecting the response for these variables, respectively.

		Mood	Num. changes when selecting mood	Sleep	Num. changes when selecting sleep	Pain	Num. changes when selecting pain	Pain level	Num. changes when selecting mood pain level
Mood	Pearson's correlation	1	−0.172^*∗∗*^	−0.133^*∗∗*^	−0.054	−0.158^*∗∗*^	−0.080	−0.335^*∗∗*^	−0.035
	Sig. (2-tailed)		0.000	0.003	0.234	0.000	0.074	0.000	0.438
	N	497	497	497	497	497	497	497	497

Num. changes when selecting mood	Pearson's correlation	−0.0172^*∗∗*^	1	0.084	0.166^*∗∗*^	−0.052	0.023	0.115^*∗*^	0.035
	Sig. (2-tailed)	0.000		0.061	0.000	0.244	0.608	0.010	0.433
	N	497	497	497	497	497	497	497	497

Sleep	Pearson's correlation	−0.133^*∗∗*^	0.084	1	0.300^*∗∗*^	0.121^*∗∗*^	0.027	0.125^*∗∗*^	0.037
	Sig. (2-tailed)	0.003	0.061		0.000	0.007	0.548	0.005	0.412
	N	497	497	497	497	497	497	497	497

Num. changes when selecting sleep	Pearson's correlation	−0.054	0.166^*∗∗*^	0.300^*∗∗*^	1	0.053	−0.025	0.139^*∗∗*^	0.402^*∗∗*^
	Sig. (2-tailed)	0.234	0.000	0.000		0.234	0.581	0.002	0.000
	N	497	497	497	497	497	497	497	497

Pain	Pearson's correlation	−0.158^*∗∗*^	−0.052	0.121^*∗∗*^	0.053	1	0.194^*∗∗*^	0.703^*∗∗*^	0.393^*∗∗*^
	Sig. (2-tailed)	0.000	0.244	0.007	0.234		0.000	0.000	0.000
	N	497	497	497	497	497	497	497	497

Num. changes when selecting pain	Pearson's correlation	−0.080	0.023	0.027	−0.025	0.194^*∗∗*^	1	0.149^*∗∗*^	0.049
	Sig. (2-tailed)	0.074	0.608	0.548	0.581	0.000		0.001	0.274
	N	497	497	497	497	497	497	497	497

Pain level	Pearson's correlation	−0.335^*∗∗*^	0.115^*∗*^	0.125^*∗∗*^	0.139^*∗∗*^	0.703^*∗∗*^	0.149^*∗∗*^	1	0.371^*∗∗*^
	Sig. (2-tailed)	0.000	0.010	0.005	0.002	0.000	0.001		0.000
	N	497	497	497	497	497	497	497	497

Num. changes when selecting mood pain level	Pearson's correlation	−0.035	0.035	0.037	0.402^*∗∗*^	0.393^*∗∗*^	0.049	0.371^*∗∗*^	1
	Sig. (2-tailed)	0.438	0.433	0.412	0.000	0.000	0.274	0.000	
	N	497	497	497	497	497	497	497	497

^*∗∗*^Correlation is significant at the 0.01 level (2-tailed). ^*∗*^Correlation is significant at the 0.05 level (2-tailed).

**Table 6:** Pearson's correlation test between survey time and user variables.

		Survey time	Mood	Sleep	Pain	Pain level
Survey time	Pearson's correlation	1	−0.049	0.007	0.335^*∗∗*^	0.323^*∗∗*^
	Sig. (2-tailed)		0.272	0.874	0.000	0.000
	N	497	497	497	497	497

Mood	Pearson's correlation	−0.049	1	−0.133^*∗∗*^	−0.158^*∗∗*^	−0.335^*∗∗*^
	Sig. (2-tailed)	0.272		0.003	0.000	0.000
	N	497	497	497	497	497

Sleep	Pearson's correlation	0.007	−0.133^*∗∗*^	1	0.121^*∗∗*^	0.125^*∗∗*^
	Sig. (2-tailed)	0.874	0.003		0.007	0.005
	N	497	497	497	497	497

Pain	Pearson's correlation	0.335^*∗∗*^	−0.158^*∗∗*^	0.121^*∗∗*^	1	0.703^*∗∗*^
	Sig. (2-tailed)	0.000	0.000	0.007		0.000
	N	497	497	497	497	497

Pain level	Pearson's correlation	0.323^*∗∗*^	−0.335^*∗∗*^	0.125^*∗∗*^	0.703^*∗∗*^	1
	Sig. (2-tailed)	0.000	0.000	0.005	0.000	
	N	497	497	497	497	497

^*∗∗*^Correlation is significant at the 0.01 level (2-tailed).

## Data Availability

The data are not available to avoid privacy infringement.
